# Cost-Effectiveness of Peer Counselling for the Promotion of Exclusive Breastfeeding in Uganda

**DOI:** 10.1371/journal.pone.0142718

**Published:** 2015-11-30

**Authors:** Lumbwe Chola, Lars T. Fadnes, Ingunn M. S. Engebretsen, Lungiswa Nkonki, Victoria Nankabirwa, Halvor Sommerfelt, James K. Tumwine, Thorkild Tylleskar, Bjarne Robberstad, PROMISE-EBF Study Group

**Affiliations:** 1 Population Health, Health Systems and Innovation (PHHSI), Human Sciences Research Council, Pretoria, South Africa; 2 School of Public Health, Faculty of Health Sciences, University of the Witwatersrand, Johannesburg, South Africa; 3 Centre for Intervention Science in Maternal and Child Health, Centre for International Health, University of Bergen, Box 7804, N-5020, Bergen, Norway; 4 Department of Clinical Dentistry, University of Bergen, Bergen, Norway; 5 Health Systems and Services Research Unit, Division of Community Health, University of Stellenbosch, Cape Town, South Africa; 6 Department of Paediatrics and Child Health, School of Medicine, Makerere University, Box 7072, Kampala, Uganda; 7 Department of International Public Health, Norwegian Institute of Public Health, Oslo, Norway; TNO, NETHERLANDS

## Abstract

**Background:**

Community based breastfeeding promotion programmes have been shown to be effective in increasing breastfeeding prevalence. However, there is limited data on the cost-effectiveness of these programmes in sub-Saharan Africa. This paper evaluates the cost-effectiveness of a breastfeeding promotion intervention targeting mothers and their 0 to 6 month old children.

**Methods:**

Data were obtained from a community randomized trial conducted in Uganda between 2006–2008, and supplemented with evidence from several studies in sub-Saharan Africa. In the trial, peer counselling was offered to women in intervention clusters. In the control and intervention clusters, women could access standard health facility breastfeeding promotion services (HFP). Thus, two methods of breastfeeding promotion were compared: community based peer counselling (in addition to HFP) and standard HFP alone. A Markov model was used to calculate incremental cost-effectiveness ratios between the two strategies. The model estimated changes in breastfeeding prevalence and disability adjusted life years. Costs were estimated from a provider perspective. Uncertainty around the results was characterized using one-way sensitivity analyses and a probabilistic sensitivity analysis.

**Findings:**

Peer counselling more than doubled the breastfeeding prevalence as reported by mothers, but there was no observable impact on diarrhoea prevalence. Estimated incremental cost-effectiveness ratios were US$68 per month of exclusive or predominant breastfeeding and U$11,353 per disability adjusted life year (DALY) averted. The findings were robust to parameter variations in the sensitivity analyses

**Conclusions:**

Our strategy to promote community based peer counselling is unlikely to be cost-effective in reducing diarrhoea prevalence and mortality in Uganda, because its cost per DALY averted far exceeds the commonly assumed willingness-to-pay threshold of three times Uganda’s GDP per capita (US$1653). However, since the intervention significantly increases prevalence of exclusive or predominant breastfeeding, it could be adopted in Uganda if benefits other than reducing the occurrence of diarrhoea are believed to be important.

## Introduction

Breastfeeding is vital to child survival, as it provides protection against diseases, including diarrhoea and acute respiratory infections [1–3]. Breastfeeding promotion is therefore central to infant health promotion in low and middle income countries (LMICS). The World Health Organisation (WHO) recommends exclusive breastfeeding (EBF) for the first six months [4]. Though breastfeeding is widespread in most LMICS, EBF is less common [5]. Globally, early initiation of breastfeeding averages about 43%, and EBF at six months is close to 39%; only a third of African children below six months are exclusively breastfed [6]. In Uganda, EBF at six months was 63% in 2011 [7].

Interventions to educate mothers on the importance of breastfeeding can increase EBF prevalence [8]. EBF promotion is mainly undertaken at health facilities, through initiatives such as the Baby Friendly Hospital Initiative and the Integrated Management of Childhood Illness [9, 10]. Community-based strategies are also reported to be efficacious [11–14]. We previously reported the results of a trial (PROMISE-EBF), where peer counselling substantially increased breastfeeding prevalence [11].

Community-based breastfeeding initiatives are rarely undertaken on a large scale in sub-Saharan Africa, and there is limited data on economic impact. In earlier publications, we presented the costs of breastfeeding promotion in Uganda [15] and South Africa [16]. The only other cost analysis of a breastfeeding intervention in sub-Saharan Africa [17] showed that community-based peer counselling could increase EBF prevalence at five months by over 70%; costs ranged from US$23 to US$126 per month of EBF [17].

In this paper, we estimate the cost-effectiveness of community-based peer counselling to promote EBF, for the reduction of diarrhoeal morbidity and mortality in Uganda, compared to facility-based breastfeeding promotion. We assess the additional benefits that can be gained from implementing a community-based breastfeeding strategy, and the associated incremental costs. This economic evaluation is based on the cluster randomised PROMISE-EBF trial, which was undertaken in Uganda between January 2006 and June 2008. The findings of this study can potentially influence investments to improve breastfeeding promotion interventions in Uganda.

## The PROMISE-EBF Trial

PROMISE-EBF was a multi-site cluster-randomised trial conducted in Burkina Faso, South Africa, Uganda, and Zambia. The aim of the study was to assess the impact of individual home-based peer counselling to promote exclusive breastfeeding for 6 months after birth. The PROMISE-EBF trial is registered with ClinicalTrials.gov, number NCT00397150. The protocol for this is available as supporting information (see the trial protocol in S1 Protocol). The trial design and results have been published elsewhere [11], and are summarised in this paper.

### Study setting and design

In Uganda, the study was undertaken in Mbale district, with a population of about 400,000 [18]. The unit of randomization were clusters of villages with an average of 1,000 inhabitants, with a birth rate of approximately 35 per cluster. Twenty-four clusters in Mbale district were selected and randomized 1:1 to either control or intervention groups, based on similarities in terms of location, urban-rural set ups and socio-economic status. Women in intervention clusters received home-based individual peer counselling from lay counsellors, to encourage EBF for six month. The women received five visits, once in the third trimester and during the first week of birth, and in weeks four, seven and ten. Extra visits were offered to mothers with breastfeeding problems or if the mother or counsellor for other reasons deemed it necessary. In the control group, women received standard care provided at public health facilities.

### Selection of peer supporters

Sensitization workshops and meetings aimed at introducing the project to community leaders were held prior to commencement of the trial. The community leaders facilitated the mobilisation of local women to work as peer counsellors on the project. Twelve women were selected as peer supporters, one in each cluster.

### Data collection

Study questionnaires were developed and adapted from previous work undertaken in the countries that participated in the trial [19–22], and were piloted in Uganda in 2005 [23]. Data were gathered between 2006 and 2008 in the mothers’ homes by trained data gatherers. The questionnaire was administered at 3, 6, 12 and 24 weeks postpartum, to determine their children’s well-being, including the breastfeeding and diarrhoea status.

### Ethics considerations

Study approval was obtained from Faculty of Medicine, Makerere University; Research and Ethics Committee, Uganda National Council for Science and Technology; and regional committees for Medical and Health Research Ethics in Norway. Women provided verbal informed consent in the peer counselling programme and signed or thumb-printed informed consent during data gathering. The age eligibility was 15 years and older. Mothers younger than 18 years had witnessed informed consent.

### Data analysis

The primary trial outcome trial was EBF prevalence and diarrhoea at 12 and 24 weeks, as reported by mothers. Current breastfeeding was assessed at all post-partum visits using a mother’s 24-hour and 7-day recall. These recall periods are widely used [7] and have been shown to provide reliable estimates [23, 24]. Children who did not receive any other food or liquids other than breastmilk were deemed to be exclusively breastfed, even if they had been administered drugs. Diarrhoea prevalence was also based on the mothers’ reports and was assessed using a 2-week recall [11], considering that shorter recall periods could provide underestimates when symptoms fluctuate from day-to-day [25]. Data analysis was done on an intention to treat basis and included all mother-infant pairs in the analysis, thus all missing, loss to follow-up and deaths were recoded as non-events (not having EBF or diarrhoea). Analyses excluding cases without valid results were also provided [11].

### Study outcomes

**Fig 1:**
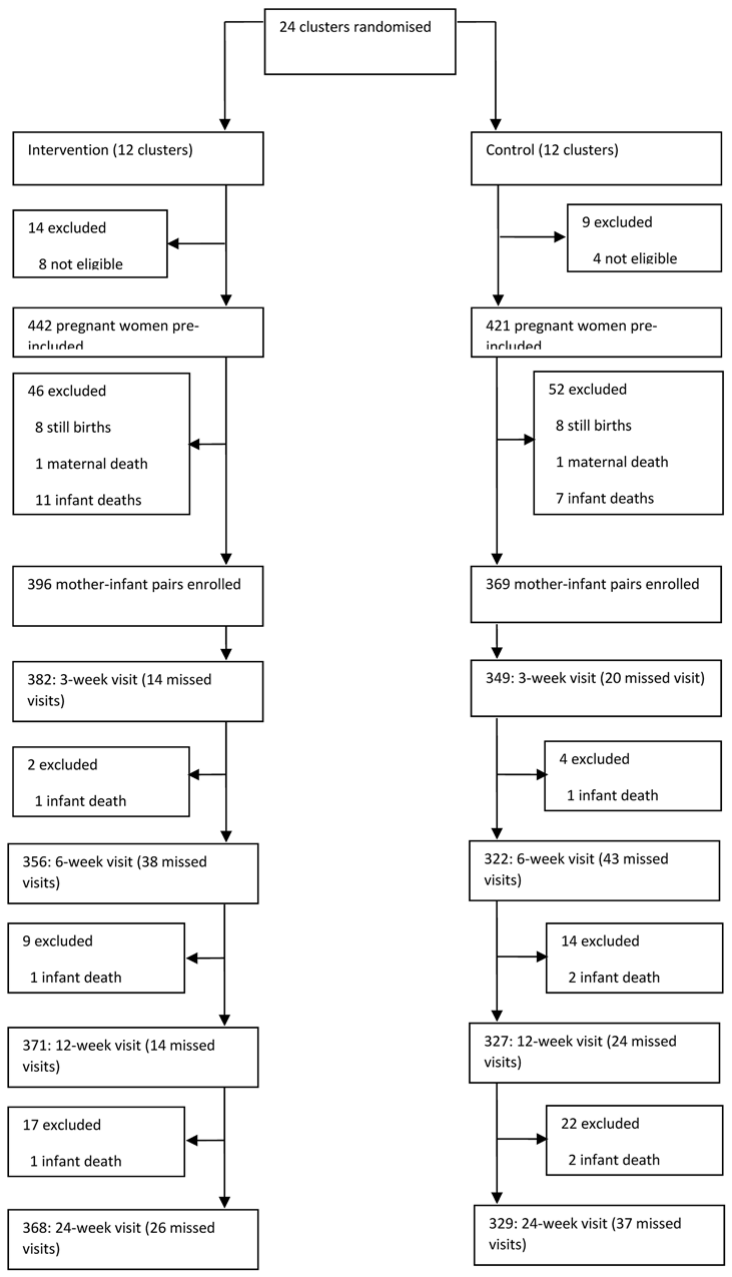
Uganda PROMISE-EBF trial profile.

A total of 329 mother-infant pairs in the intervention and 368 in the control group were followed up until 24 weeks after birth (Fig 1). At 12 weeks, EBF prevalence (24 hour recall) in the intervention group was 82%, compared to 44% in the control group, with a prevalence ratio (confidence interval) of 1.89 (1.70–2.11) [11]. The 12 week diarrhoea prevalence was 10% in the intervention and 9% in the control group, prevalence ratio 1.13 (0.81–1.59). Other factors, including socio-economic status, maternal age, parity, mothers’ education and marital status were fairly evenly distributed between intervention and control groups, and were not deemed to affect breastfeeding and diarrhoea outcomes [11].

## Methods for the Economic Evaluation

An economic model based on the PROMISE-EBF trial was created in order to estimate the cost-effectiveness of using peer counselling to promote exclusive breastfeeding. Because some of the data needed to populate the model, such as hospitalisation rates and disease costs, were not captured during the trial, this was supplemented by evidence from the literature. The CHEERS checklist was used to guide reporting (see the checklist in S1 Checklist).

### Comparators in the economic evaluation

The economic evaluation compares two breastfeeding promotion strategies, based on the control and intervention groups in the trial: 1) community-based peer counselling conducted alongside breastfeeding promotion in facility-based maternal and child health services, including antenatal and postnatal services (herein after referred to as *peer counselling*); and 2) breastfeeding promotion in facility-based maternal and child health services only (herein after referred to as *health facility breastfeeding promotion—HFP*). This followed the set-up in the PROMISE-EBF trial, where peer counselling was offered to pregnant women and mothers of newborns, while in both control and intervention clusters, women could access standard health facility services.

Facility-based breastfeeding promotion is often done at antenatal and postnatal services. Many facility-based maternal and child health packages provide health advice and other services for improved maternal and child health. Women are encouraged to attend antenatal clinics at least once during pregnancy and other under-five services after birth. Over 90% of pregnant women in Uganda access antenatal care (ANC) at least once before or after delivery, and over 50% attend postnatal services [7]. PROMISE-EBF did not assess the quantity or quality of facility-based services, but parallel research within our study group has described current practices in the study setting [26].

### The economic model

**Fig 2:**
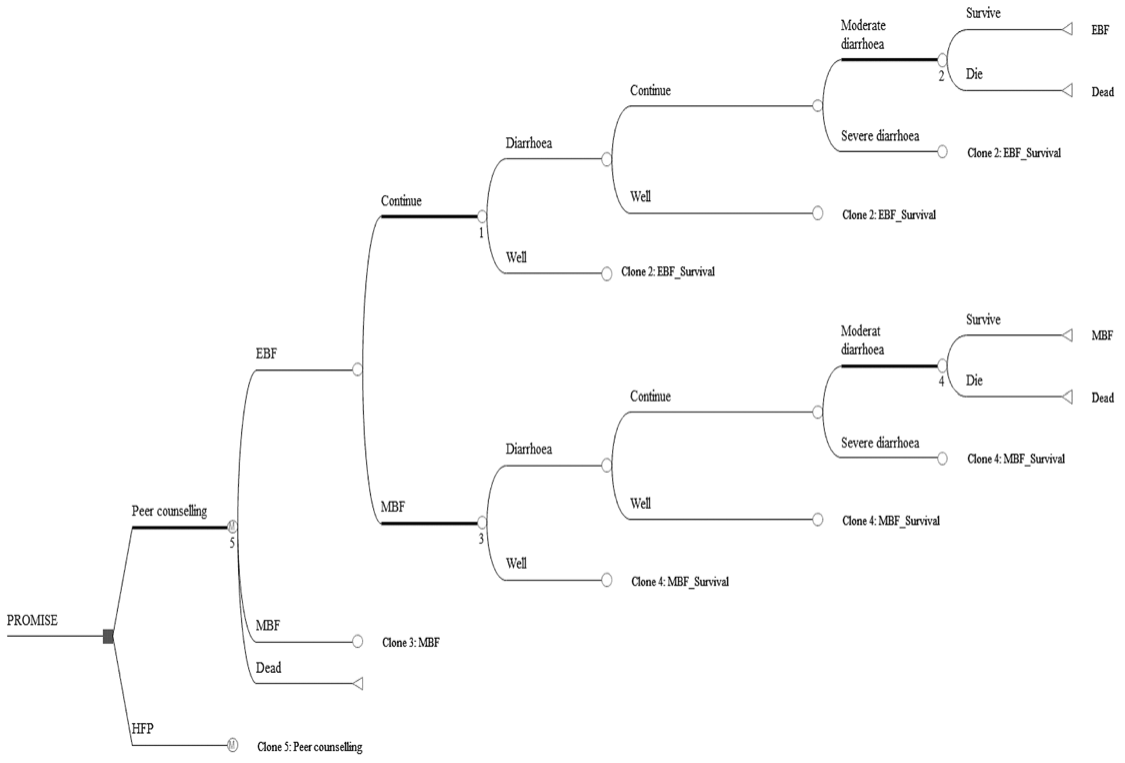
Decision model of cost-effectiveness analysis for peer counselling vs HFP.

A decision tree with a state Markov model was used to depict breastfeeding promotion and the associated feeding patterns in the first six months of life (Fig 2). The model predicts feeding patterns and children can have one of two options: exclusive or predominant breastfeeding (EBF/PBF) and mixed or replacement feeding (MF/RF), see Box 1 for definitions. The merging of EBF and PBF was done because few studies indicate clear differences in health outcomes between EBF and PBF [11, 27]. MF and RF were merged because only 0.3% of the children in the PROMISE-EBF trial received replacement feeding. In the model, children were allowed to move both ways between feeding states. This was done to reflect the findings of the PROMISE-EBF trial, which showed that mothers tended to periodically switch between EBF/PBF and MF [28]. This might be the case, since the definition of EBF/PBF was based on shorter periods (24-hours and 1-week recall), which may allow for more frequent fluctuations and may under-represent usual practice [29].

**Box 1. Definitions of infant feeding patterns.**

**Exclusive breastfeeding** is when only breast milk is given to the child, except for medicines, vitamins or mineral supplements.

**Predominant breastfeeding** is when breast milk is nutritionally dominant while given in addition to water-based fluids including fruit juices, tea without milk or oral rehydration salts.

**Complementary feeding including breast milk (partial breastfeeding or mixed feeding)**: These terms are used to describe when non-human milk, semi-solids or other solids are given to the child in addition to breast milk. The term mixed feeding does normally refer to the feeding practice specified above during the first half of infancy (under 6 months old).

**Replacement feeding** is defined as the feeding strategy when breastfeeding has been stopped, or if the child never has been given any breast milk. Exclusive replacement feeding was defined as when never having given any breast milk.

At each feeding state a decision tree model depicts three health scenarios: sick (diarrhoea), well (no diarrhoea) and dead. The diarrhoea state could either be severe, in which case the child would be hospitalised, moderate–requiring outpatient treatment, or mild–requiring no treatment. Probabilities of diarrhoea and mortality were assumed to depend on the feeding practice, i.e. whether a child belonged to the EBF/PBF or MF/RF pattern.

The model had monthly cycles, terminating at six months, based on the PROMISE-EBF trial. It was based on the hypothesis that additional breastfeeding promotion increases EBF/PBF prevalence, which in turn could affect morbidity and mortality.

### Model parameters

#### EBF and diarrhoea prevalence

In the PROMISE-EBF trial, mothers were followed up for six months, and a questionnaire was administered at 3, 6, 12 and 24 weeks postpartum. These data were used to estimate feeding and diarrhoea state transition probabilities.

Feeding state transition probabilities were estimated using parametric survival models [30] of EBF/PBF duration (Table 1) [28]. A multiple events model was used to estimate survival. The event was cessation of EBF/PBF, a time dependent variable indicating the age at which EBF/PBF was stopped. Cases were censored if respondents were lost to follow-up, missed the last valid interview, or continued EBF/PBF beyond six months.

**Table 1:** Transition probabilities between feeding states in the PROMISE trial.

Months	Control				Intervention			
	*α*_1_	*α*_2_	*α*_3_	*α*_4_	*α*_1_	*α*_2_	*α*_3_	*α*_4_
1	0.67	0.33	0.82	0.18	0.81	0.19	0.93	0.07
2	0.73	0.27	0.76	0.24	0.89	0.11	0.85	0.15
3	0.74	0.26	0.72	0.28	0.91	0.09	0.79	0.21
4	0.75	0.25	0.70	0.30	0.92	0.08	0.75	0.25
5	0.76	0.24	0.68	0.32	0.92	0.08	0.71	0.29
6	0.77	0.23	0.66	0.34	0.93	0.07	0.68	0.32

Source: Chola et al. 2013. *Infant feeding transitions among Ugandan children from the cluster-randomised trial PROMISE-EBF*.

*α*_1_ = Probability of remaining in EBF/PBF; *α*_2_ = Probability of transitioning from EBF/PBF to MF/RF; *α*_3_ = Probability of remaining in MF/RF; *α*_4_ = Probability of transitioning from MF/RF to EBF/PBF.

Probabilities of diarrhoea events (sick or well) were estimated using longitudinal/panel methods in STATA. The aim was to assess diarrhoea morbidity in infants during the six month follow-up. We estimated four transition probabilities between two states (sick and well) as shown in Table 2. The events were dependent on a child’s feeding state.

**Table 2:** Transition probabilities between health states in the PROMISE-EBF trial.

	*β*_1_	*β*_2_	*β*_3_	*β*_4_
Intervention group				
EBF/PBF	0.08	0.88	0.12	0.92
MF/RF	0.07	0.86	0.14	0.93
Control group				
EBF/PBF	0.06	0.64	0.36	0.94
MF/RF	0.10	0.71	0.29	0.90

*β*_1_ = Probability to be sick with diarrhoea in respective visit and continued diarrhoea in next visit; *β*_2_ = Probability of diarrhoea in respective visit and changed to well (no diarrhoea) in next visit; *β*_3_ = Probability of being well in respective visit, and continue in this state in next visit; *β*_4_ = Probability of well in respective visit and sick in next visit.

#### Hospitalisation for diarrhoea

The PROMISE-EBF trial did not collect data on diarrhoea hospitalisation. We based our assumptions on diarrhoea hospitalisation on a systematic review of breastfeeding and the risk of diarrhoea and diarrheal death [31]. This study reviewed literature published from 1980 to 2009 on suboptimal breastfeeding and the associated diarrhoea morbidity and mortality. Random effects meta-analyses were conducted on 18 studies, to generate pooled relative risks of diarrhoea hospitalisation and death. The estimated relative risk (95% confidence interval) of diarrhoea hospitalisation was 2.3 (0.08–6.55) for predominantly, 4.4 (1.75–13.84) for partially and 19.5 (6.13–33.86) for not breastfed infants 0–5 months of age, compared to those exclusively breastfed (Table 3).

**Table 3:** Base parameters used in the decision model for exclusive breastfeeding promotion.

Parameter	Estimate		Source
Mortality rates	Prevalence	SA range	
Control	3/1000	0.001–0.01	PROMISE-EBF
Intervention	8/1000	0.001–0.01	PROMISE-EBF
Hospitalisation	Relative risk	95%CI	
Predominantly breastfed	2.28	(0.08–6.55)	Lamberti *et al*. (2011)
Partially breastfed	4.43	(1.75–13.84)	Lamberti *et al*. (2011)
Not breastfed	14.40	(6.13–33.86)	Lamberti *et al*. (2011)
Costs (US$)	Mean	SA range	
Health facility promotion per child per year	3.60	2.20–6.43	Orach et al (2003)/Levin et al (2007)
Peer counselling per child per year	139	74–233	Chola et al (2011)
Hospitalised diarrhoea per case	95	65–134	Chola & Robberstad (2009), Aikins et al (2010), Tate et al (2009)
Non-hospitalised diarrhoea per case	9	3.80–26	Chola & Robberstad (2009), Aikins et al (2010), Tate et al (2009)
Other parameters	Estimate	SA range	
Discount rate (r)	0.03	0–0.06	
Life expectancy (years)	53	38–77	WHO (2014)
Diarrhoea disability weight			Murray et al (2012)
Mild	0.061		
Moderate	0.202		
Severe	0.281		
Age at onset (years)	0.1		Own assumption
Mean duration of diarrhoea (days)	7		Kirkwood et al (1991)

CI = Confidence interval; RR = Relative risk; SA = Sensitivity analysis.

#### Cost estimates

Costs were assessed from a provider perspective (Table 3), mainly because the cost analysis done on the PROMISE-EBF trial was retrospective and only costs from project accounts could be obtained [15].

Costs of peer counselling were estimated in the PROMISE-EBF trial. The structure of the trial could be broken in four basic functions: administration; peer supervision and coordination; peer support; and recruitment (done by recruiters, who recruited mothers into the trial. These were not peer supporters). We thus used an ingredients approach to costing, in order to capture the different elements of the trial. Costs included capital items such as motor vehicles, furniture and computers; and recurrent items such as salaries, fuel and rentals. Staff costs were based on the local rates paid to employees in the trial. Peer supporters and recruiters were not permanent members of staff. They were offered a token US$20 every month for their participation, a figure that was arrived at in a meeting with peer supporters, where they agreed on this as adequate compensation for their time. Intervention start-up costs, as well as costs of training and retraining of peer supporters were also included in the analysis. Detailed descriptions and analyses of the costs of the PROMISE-EBF trial in Uganda are given elsewhere [15]. Annual intervention costs were estimated to be US$56,308, and the cost per mother/infant pair (the output required in the economic model) was US$139 per year. The cost per visit was US$26 and the cost per week of EBF was estimated to be US$15 at 12 weeks post-partum.

Costs of HFP were not collected in the PROMISE-EBF trial, but were averaged from results of two studies assessing maternal health services. Orach *et al*.[32] estimated an average cost of antenatal and postnatal services ranging from US$2 to US$6 per child in three rural districts in Uganda. Levin *et al*. [33] estimated an average antenatal and postnatal services cost between US$2 to US$4 per child in Uganda. The average cost of US$3.6 was used in the model.

Diarrhoea treatment costs were also not collected in the trial. These were estimated form *ad hoc* and PubMed searches for articles in English undertaken in the last decade on treatment costs of diarrhoeal disease among infants in sub-Saharan Africa. The search words were costs, cost analysis and diarrhoea. The criteria for selection were that studies presented results from a provider perspective and provided costs for children less than five years. Studies were considered if costs were provided for outpatient, inpatient care or both. The search yielded 321 articles, whose abstracts were individually scrutinised. Fifteen articles were then selected for full text assessment, out of which 3 were finally included from Kenya [34], Ghana [35] and Zambia [36] (Table 3).

All costs were converted to 2007 prices using the local (specific country) consumer price index, and then converted to US$ at the estimated exchange rate in 2007.

#### Effect measures

Two measures of effectiveness were calculated as follows:

1. Person months of EBF/PBF extracted from PROMISE-EBF data–estimated as the cumulative months of EBF/PBF (MEBF). The mean duration of EBF/PBF estimated from the survival analysis was 1.5 in the control (HFP) and 3.5 months in the intervention (peer counselling) group [28].
2. Disability adjusted life years (DALYs) due to diarrhoea–calculated as years of life lived with disability (YLD), added to years of life lost (YLL) due to diarrhoea death. YLLs were calculated assuming a mean duration of seven days [37] average age at onset of 0.1 years, disability weights of 0.061 for mild, 0.202 for moderate and 0.281 for severe diarrhoea [38] and life expectancy at birth of 53 years [39]. Mortality estimates were made using PROMISE-EBF data. The estimated all-cause mortality rates were 3/1000 children in the control and 8/1000 children in the intervention group [11]. DALYs were not age-weighted, but discounted at a rate of 3% per annum.

### Sensitivity analyses

Parameter uncertainty was assessed in one way sensitivity analyses. Life expectancy was varied from 38 years (males in Sierra Leone) to 77 years (females in Libya) [39]. Discount rates for both costs and outcomes were varied from 0% to 6% as recommended in the literature [40]. Disease costs were varied as follows: US$3.8 to US$26 for non-hospitalised and US$65 to US$134 for hospitalised diarrhoea cases, based on the maximum and minimum ranges of costs found in literature [34–36]. Diarrhoea hospitalisation rates were varied according to the upper and lower limits of the confidence intervals of the respective relative risk [31]. Costs of peer counselling were varied from US$74 to US$233. The low value was obtained from a scenario analysis performed in the costing study [15] and the high value from a similar study estimating the costs of peer counselling in Zambia (data available on request). A range of US$2.2 to US$6.4 was adopted for costs of HFP, representing the lowest and highest costs identified in the literature [32, 33].

Probabilistic sensitivity analyses were also undertaken. Beta distributions were fitted for probability parameters using point estimates and standard errors. Lognormal distributions were fitted for relative risks using point estimates and their confidence intervals. The gamma distribution was used for costs and DALYs, with mean costs also representing the standard error [30].

### Statistical analyses

We used Microsoft Excel to summarise cost data, STATA version 11 for statistical analyses and TreeAge Pro 2012 Suite to model cost effectiveness.

## Results

### Base case cost-effectiveness

The base case results are presented in Table 4. The costs of breastfeeding promotion were US$112 for HFP and US$249 for peer counselling, with an incremental cost between the interventions of US$137.

**Table 4:** Base case results of the cost-effectiveness analysis.

CEA of increasing EBF prevalence						
**Cost per MEBF**						
Strategy	Cost (US$/Child)	IC	MEBF/Child	IE	Cost/MEBF	ICER
HFP	113		1.50		75	
Peer counselling	250	137	3.50	2.00	71	68
**Cost per DALYs averted**						
Strategy	Cost (US$/Child)	IC	DALY/Child	DALYs	Cost/DALY	ICER
				averted		
HFP	113		5.76		19	
Peer counselling	250	137	5.78	0.01	43	11,353

IC = Incremental cost; IE = Incremental effect; DALYs = Disability adjusted life years; MEBF = Months of EBF; ICER = Incremental cost effectiveness ratio (IC/IE). US$ = United States Dollars.

The expected cumulative period of EBF was longer for mothers receiving peer counselling (3.5 months) than for mothers in the control group who relied on HFP alone (1.5 months) [28]. The associated incremental effectiveness was two months. The average cost per MEBF was US$75 for HFP and US$71 for peer counselling, with an incremental cost-effectiveness ratio of US$68, in favour of peer counselling.

The difference in DALYs averted for HFP and peer counselling was negligible. Irrespective of intervention, the health loss attributable to morbidity and mortality is about five DALYs per child, with negligible difference of 0.01 in favour of peer counselling. The incremental cost-effectiveness ratio of peer counselling is US$11,353 compared to HFP.

### Sensitivity analysis

**Fig 3:**
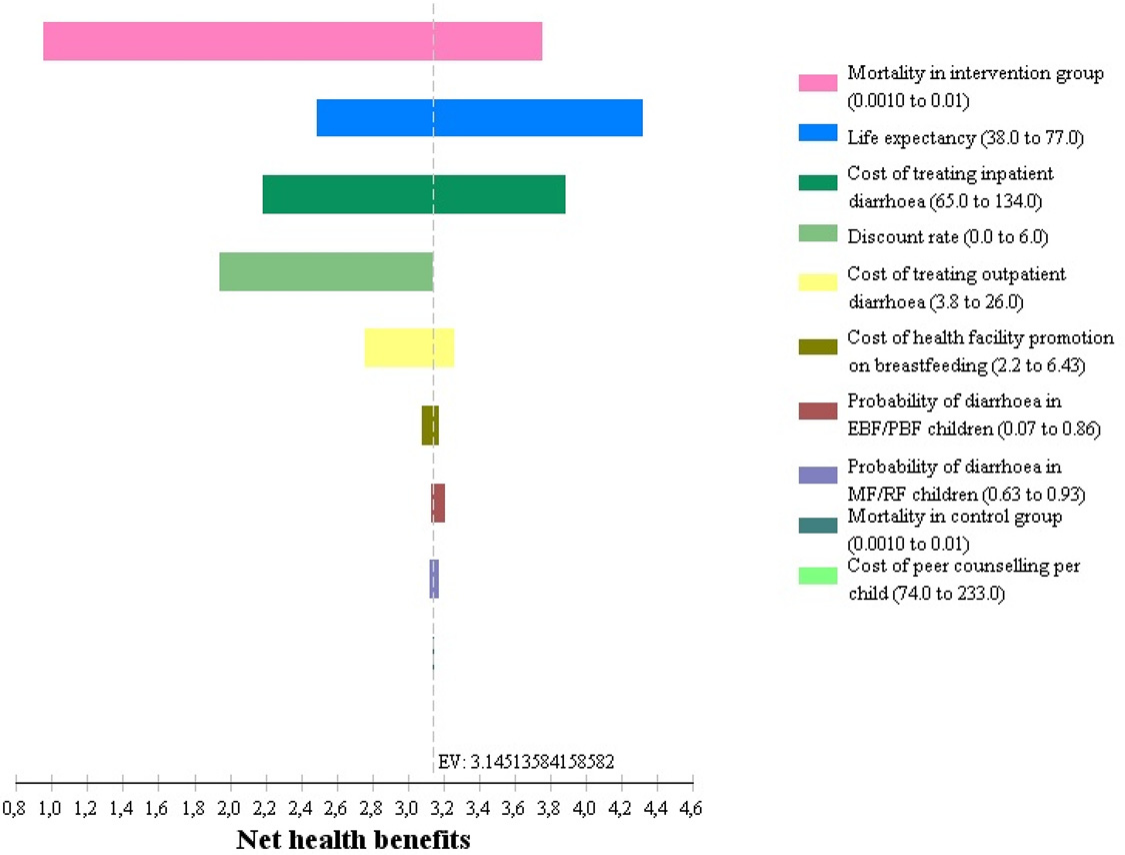
Tornado diagram of one-way sensitivity analyses results of uncertain model parameters. EV = expected value. Net health benefits = ((Effectiveness–Costs)/Willingness to pay).

As shown in the tornado diagram in Fig 3, the mortality rate in the intervention group had the greatest impact, accounting for 49% of the uncertainty, followed by life expectancy (21%), and cost of treating severe diarrhoea (18%). A further 10% of the variation was accounted for by the discount rate (9%) and the cost of treating moderate diarrhoea (1%). Varying the other parameters had little impact on cost-effectiveness.

**Fig 4:**
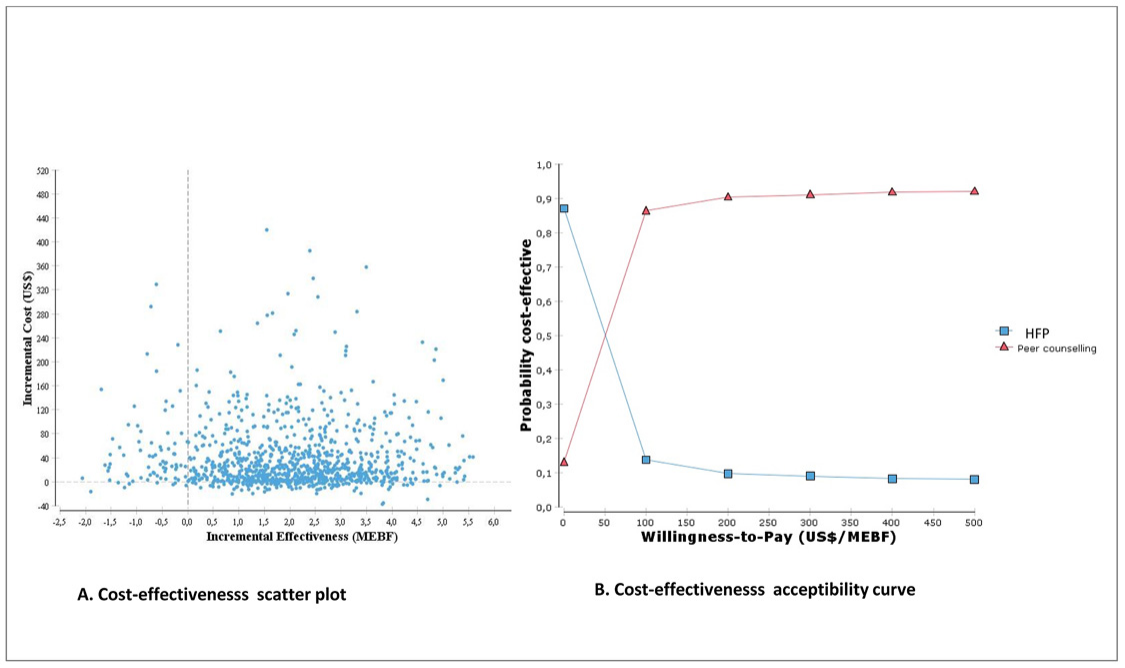
Cost-effectiveness scatterplot and acceptability curves for peer counselling vs HFP (Cost/MEBF).

**Fig 5:**
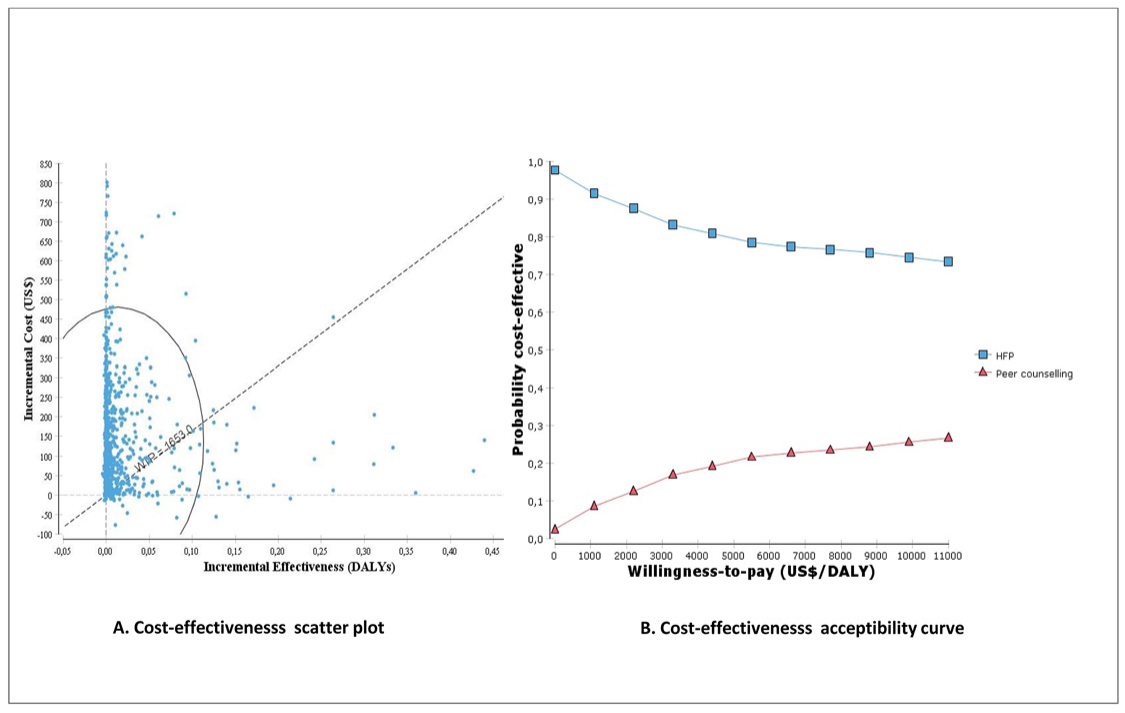
Cost-effectiveness scatterplot and acceptability curves for peer counselling vs HFP (Cost/DALY).

Results from probabilistic sensitivity analyses are illustrated in Figs 4 and 5. Fig 4 shows the scatter plot and incremental cost-effectiveness acceptability curve of peer counselling vs HFP for the MEBF outcome. Fig 4A illustrates how the uncertainty is distributed across both costs and effectiveness, while Fig 4B, shows that peer counselling has a higher likelihood of being cost-effective compared to HFP if willingness-to-pay exceeds US$68 per MEBF.

Fig 5 shows the incremental cost-effectiveness scatterplot and acceptability curves of peer counselling vs HFP for DALYs. The scatter plot (Fig 5A) shows that peer counselling is not likely to be a cost-effective strategy in averting DALYs. Only 9% of the ICERs in the scatter plot are cost-effective i.e. fall below a willingness-to-pay threshold of US$1653 per DALY (about 3 times Uganda’s per capita GDP of US$551 [41]). In Fig 5B, it is shown that HFP is more likely to be cost-effective than peer counselling.

## Discussion

This study is the first economic evaluation of a breastfeeding intervention in Uganda and the second such study in sub-Saharan Africa [17]. Peer counselling is substantially more costly than standard facility-based services alone, and is only likely to be considered cost-effective if the willingness-to-pay to increase breastfeeding prevalence exceeds US$68 per MEBF, or US$11,353 per DALY averted. Uganda has a number of pending maternal and child health interventions with low coverage that are far more cost-effective.

Results from the PROMISE-EBF trial indicate that there was no association between increased breastfeeding prevalence and diarrhoea morbidity [11]. It should be noted that even though we calculated DALYs using trial data, the PROMISE-EBF trial was not powered to identify an effect of the intervention on mortality. A discussion on these issues is provided elsewhere [11].

### Study strengths and limitations

A major strength of this study is that we primarily use data from a randomised trial, which minimises selection bias [42]. However, the trial only compared two options: an intervention and the standard of care. Our economic evaluation therefore only compares a subset of all available options for promotion of EBF. For example, we did not consider the option of introducing group counselling, which has also proved to be effective [13].

It was out of the scope of this study to make head-to-head economic comparisons of breastfeeding promotion and other cost-effective child health interventions such as rotavirus vaccinations, diarrhoea management, zinc and vitamin A supplementation [43–45]. Since results are presented using DALYs averted, comparison is possible across a broad range of disease groups. Cautious priority considerations are therefore possible on a broader basis if the perspectives and methods of the different studies are reasonably comparable.

Furthermore, this study only addresses diarrhoea despite evidence that breastfeeding influences several illnesses, among them acute respiratory infections (ARI), otitis media and juvenile diabetes [46, 47]. In addition, mortality is influenced by factors other than feeding practice, and this was not fully addressed in this study. Our estimates of the cost-effectiveness of EBF promotion are thus conservative. In addition, we only considered the impact of breastfeeding for infants up to six months, while some evidence indicate benefits of breastfeeding well into childhood [48, 49]. This again is likely to underestimate the full effect of breastfeeding promotion interventions, which should be taken into consideration in future evaluations as the empirical evidence improves.

Limited health system cost and disease data for breastfeeding infants meant that we had to make assumptions about some parameters, especially regarding the transferability of hospitalisation and cost data. Data on the costs of different feeding modes from the perspective of the mothers and families is missing altogether. Since we did not conduct a marginal costing analysis we cannot inform the impact of changing the number of health facility visits, personnel or other resources that will have to be increased when the intervention is scaled up. Data on costs of diarrhoea are taken from studies done in other countries, which may have different cost structures from Uganda. This could underestimate or overestimate the true diarrhoea costs in Uganda. Comprehensive sensitivity analyses are conducted on this parameter.

### Comparison with other studies

Our result in terms of cost per MEBF is similar to that found in a South African study [17]. Desmond *et al*. estimate incremental costs of breastfeeding promotion ranging from US$23 to US$126 per MEBF, compared to US$68 in our study. The South African intervention was more intensive, with four home visits to mothers antenatally, compared to one in PROMISE-EBF, and 14 postnatal visits compared to four in our intervention. The mean duration of EBF in the intervention arm of the South African study was five months [17], compared to 3.5 months in ours [11]. While Desmond and colleagues conclude that EBF promotion through peer counselling is a cost-effective strategy, we are reluctant to draw this conclusion because the cost per DALY estimates were not favourable. In separate cost analyses of the PROMISE-EBF trial in South Africa and Uganda, we undertook scenario analyses to show the impact on unit costs, if the intervention was integrated with primary healthcare. The results showed that the cost per visit could US$32 in South Africa [16] and US$14 in Uganda [15].

### Policy implications

The estimated incremental cost of US$68 per MEBF is more than Uganda’s per capita health expenditure of US$43 [50]. It is difficult to conclude on the basis of this evidence whether the intervention is justified, especially since it did not seem to carry any advantages in terms of infant growth [51]. This study shows that peer counselling is likely to be the cost-effective strategy for all willingness-to-pay thresholds above US$68 per MEBF.

PROMISE-EBF was a community randomised trial, with more supervision and intensity than a comparable facility-based intervention. We are uncertain about the impact of scaling up the intervention to district or even national level over a lengthy period. However, it has been shown that community strategies may achieve better health outcomes than facility-based strategies, especially in areas with limited health facilities [52].

In the sensitivity analyses, we tested a range of values that were most likely to capture the extreme changes in parameter estimates. The most sensitive parameter was the mortality rate in the intervention group, which accounted for 49% of the uncertainty. The other parameters used in the model were less influential on the cost-effectiveness estimates. This is an important result, particularly given the scarcity of information in resource poor settings. The model used in this study can thus be used to make fairly accurate projections on the most probable costs and consequences of undertaking similar interventions in Uganda and other sub-Saharan African countries.

## Conclusions

Peer counselling to promote EBF is substantially more expensive than providing advice only through standard health facility based care. In our study, peer counselling increased both exclusive and predominant breastfeeding prevalence in the first six months. Based on our estimations, peer counselling is not likely to be cost-effective in averting DALYs associated with diarrhoea. This conclusion may be different in areas where increased EBF prevalence has been demonstrated to reduce diarrhoea prevalence more than in Uganda or if assessing other morbidity outcomes. We recommend analysis of cost-effectiveness when beneficial effects of the occurrence also of other illnesses are captured.

## Supporting Information

S1 Checklist CHEERS Checklist used for the article. (PDF)

S1 Protocol The PROMISE-EBF Trial protocol. (PDF)
